# Systematic review of digital health interventions to support self-management of low back pain in the workplace

**DOI:** 10.1177/20552076251336281

**Published:** 2025-05-26

**Authors:** Minghao Chen, Valerie Sparkes, Liba Sheeran

**Affiliations:** 1 School of Healthcare Sciences, Cardiff University, Heath Park Campus, Cardiff, UK; 2 Biomechanics and Bioengineering Research Centre Versus Arthritis, Cardiff, UK; 3 School of Health Sciences, University of Southampton, Highfield Campus, University Road, Southampton, UK

**Keywords:** Low back pain, workplace interventions, digital health, self-management, tailored approaches

## Abstract

**Background:**

Low back pain (LBP) is a prevalent condition in working populations, imposing significant individual, organisational, and societal burdens, including reduced quality of life, impaired work performance, and high healthcare costs. Digital health interventions (DHIs) offer scalable solutions for self-managing LBP in workplace settings, yet their tailoring, integration, and effectiveness remain unclear.

**Objective:**

This systematic review evaluates the effectiveness of DHIs in supporting LBP self-management in workplace environments. It examines intervention components, tailoring methods, integration with occupational health (OH) pathways, and their impact on clinical and work-related outcomes.

**Methods:**

A systematic search was conducted across PubMed, MEDLINE, EMBASE, CINAHL, the Cochrane Library, and Web of Science, following PRISMA guidelines. Randomised controlled trials evaluating DHIs for workplace LBP were included. Data extraction focused on intervention characteristics, tailoring approaches, and primary outcomes, including pain intensity, disability, and physical performance. The quality of evidence was assessed using the Cochrane risk-of-bias and GRADE frameworks.

**Results:**

Five studies were included, featuring DHIs delivered via web-based platforms or mobile applications. Interventions incorporated exercise, ergonomics education, and work activity modification. Only one study used a tailored approach based on theoretical frameworks and individualised work classifications. Moderate-quality evidence supported improvements in pain, disability, and physical performance, but effects on quality of life, psychosocial factors, and work outcomes were inconsistent. Integration with occupational health pathways was absent in all studies.

**Conclusions:**

The lack of tailoring and integration within occupational health systems limits the scalability and impact of DHIs for workplace LBP. Future research should focus on personalised, theory-driven interventions and systemic alignment with occupational health policies to enhance their feasibility, implementation, and long-term outcomes.

## Introduction

Low back pain (LBP), defined as pain occurring between the lower rib margin and the buttock creases with or without neurological symptoms in the lower limbs,^1^ is a prevalent public health issue and the leading global cause of disability.^2^ The majority of LBP is termed non-specific LBP, characterised by pain that is not attributed to any specific, identifiable pathological cause.^3^ While acute pain symptoms may resolve within 4–6 weeks, one in three individuals continue to report persistent pain 1 year after onset.^4^ LBP prevalence peaks at 42% among working-age individuals between 40 and 69 years.^5^ As the second most common cause of absenteeism, LBP leads to significant direct and indirect costs,^6^ imposing substantial burdens on individuals, organisations, and governments.^7^ In the UK, 7.8 million workdays were lost due to musculoskeletal disorders between 2023 and 2024, with LBP most prevalent.^8^ An enormous financial burden stemming from LBP has also been reported, with an estimated £3.5 billion in direct healthcare costs related to consultations and prescriptions.^9^ Within the workplace, the burden of LBP is further exacerbated, leading to presenteeism (working whilst ill), productivity losses, and reduced job satisfaction, which in turn impact staff retention and increase litigation risks.^10,11^

Workplace interventions for LBP include ergonomic adjustments, exercise programmes,^12^ and worksite training^13^ but these often target single factors and lack flexibility for diverse occupational settings limiting their effectiveness.^14^ To address these limitations, multifaceted interventions integrating ergonomic, psychological, educational, and exercise components have been developed and shown effectiveness.^15,16^ More recently, self-management interventions have gained prominence as the first line of treatment, empowering individuals to develop self-management skills and confidence in managing their condition.^17,18^ Growing research on workplace self-management, supported by several systematic reviews (SRs), highlights the potential of person-centred self-management interventions, such as participatory ergonomics^19^ and tailored exercise programmes,^20^ to improve clinical and work-related outcomes while emphasising the critical influence of the intervention tailoring to individual's and job demands, and organisational support.^12,16,19,21–26^

Digital health technologies offer an accessible and scalable approach to self-management, supporting its delivery through information technology such as computers, mobile phones, and handheld devices to deliver evidence-based support.^27^ Evidence suggests that digital health interventions (DHIs) designed to support self-management aligned with workplace representatives’ needs could bring improvements in pain and function for chronic conditions including LBP, but the included studies were not limited to workplace settings.^28^ Most research has been conducted in clinical settings, with limited evaluation taking place in workplace environments.^29,30^ In addition, the challenges remain regarding tailoring interventions to individual and workplace-specific needs, as well as their integration into existing occupational health frameworks, both of which are crucial for successful implementation and sustained impact.^31^

Therefore, this SR aims to evaluate the effectiveness of workplace-based DHIs that support self-management of LBP on health and work outcomes, examine how these interventions are tailored to individual and occupational needs, and assess their integration into workplace health pathways. Findings will provide insights to enhance the design and implementation of DHIs for workplace LBP management.

## Materials and methods

This SR was designed and reported following the Preferred Reporting Items for Systematic Reviews and Meta-Analyses (PRISMA) guidelines^32^ [Supplemental Appendix 1]. The protocol was pre-registered in PROSPERO (ID: CRD42023435184).

### Inclusion criteria

The eligibility criteria for this review were defined using the PICO framework.^33^

Studies included working populations with LBP, defined as pain between the lower rib margin and buttock creases,^1^ lasting over 4 weeks (subacute and chronic).^34^ Participants presenting red flags for serious pathology or systemic illness were excluded. The working population included individuals in full-time, part-time, or self-employed roles with no restriction on occupation type.^35^ Eligible interventions were DHIs that delivered self-management information via computers, mobile phones, or handheld devices without requiring direct feedback from healthcare professionals.^27^ Studies involving DHIs integrated into medical treatments or without pre-designed intervention content were excluded. Control groups in eligible studies could include usual care, non-digital self-management interventions, or non-tailored DHIs to support self-management. The review considered primary outcomes such as pain intensity and disability levels measured by validated scales.^30,36^ Secondary outcomes included work-related measures like absenteeism and productivity, as well as quality of life, physical performance, and psychosocial factors such as stress, anxiety, and depression. Only randomised controlled trials (RCTs) published in English were included.

### Search methods

A three-step search strategy was used to identify studies published in six databases from inception up to February 2023, with the final search conducted in March 2024. An initial pilot search of MEDLINE was conducted to refine search terms. Subsequently, a comprehensive search was carried out in PubMed, MEDLINE, EMBASE, CINAHL, the Cochrane Library, and Web of Science. The search terms were grouped into three concepts: (1) LBP, (2) DHIs, and (3) workplace [Supplemental Appendix 2]. Additional searches included reference lists from relevant reviews, unpublished theses, conference papers, and trial registrations to ensure comprehensive coverage.

### Data collection, analysis, and outcomes

All identified studies were uploaded to Rayyan for screening and analysis.^37^ Titles and abstracts were screened independently by two reviewers (MC and LS), with discrepancies resolved through consultation with a third reviewer (VS). Data from included studies were extracted into an Excel spreadsheet, covering study characteristics (e.g. publication date, population demographics, and intervention details) and outcomes (e.g. pain, disability, and work-related measures). Due to heterogeneity in intervention content, control settings, and outcome measures, a meta-analysis was deemed inappropriate. Instead, a narrative synthesis was used to describe the study findings.^38^ Given the moderate to high risk of bias and substantial heterogeneity across studies,^39,40^ statistical pooling through meta-analysis was not conducted. Descriptive tables summarised study characteristics, and narrative synthesis provided insights into intervention effects on primary and secondary outcomes.

### Risk of bias assessment

The risk of bias in included studies was assessed using the Cochrane Risk of Bias Tool,^41^ evaluating factors such as sequence generation, allocation concealment, blinding, incomplete data management, selective reporting, and other potential biases. Two reviewers (MC and LS) independently assessed the studies, with disagreements resolved by a third reviewer (VS).

### Quality assessment

To assess the quality and strength of the evidence, the GRADE (Grading of Recommendations Assessment, Development, and Evaluation) framework was applied. This involved evaluating five factors – risk of bias, inconsistency, indirectness, imprecision, and publication bias – for each outcome, categorising them as not serious, serious, or very serious. Outcomes from RCTs were initially rated as high-quality evidence but could be downgraded to moderate, low, or very low quality based on these criteria. This comprehensive approach ensured a nuanced interpretation of the findings within the context of the evidence's strengths and limitations. Two reviewers carried out the quality assessment independently, with any disagreements resolved through discussion or consultation with a third reviewer.

## Results

### Results of the search

**Figure 1.**
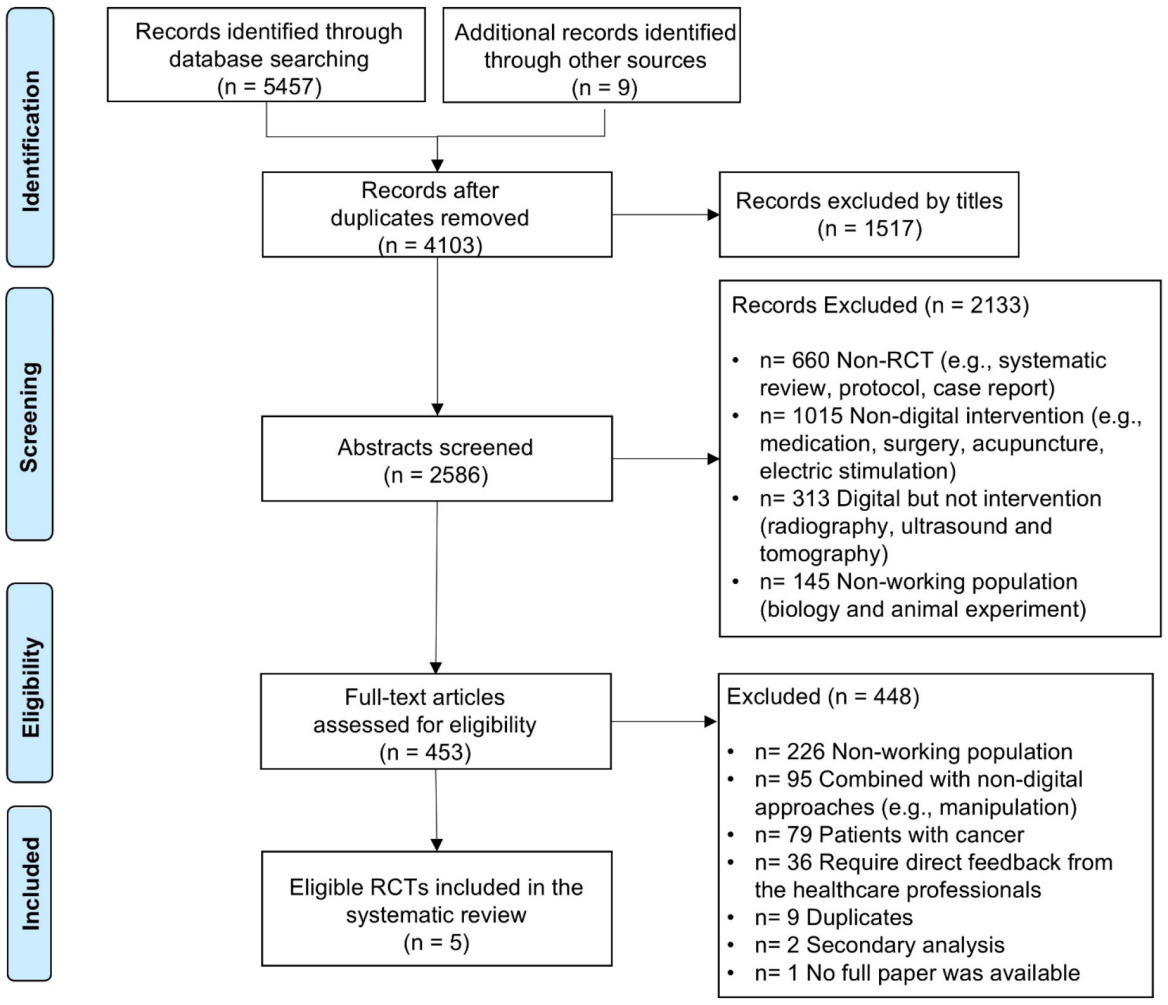
Flow diagram of the search strategy and selection process using preferred reporting items for systematic reviews (PRISMA).

An initial search of six major databases yielded 5457 articles, with an additional nine studies identified from other sources. After removing duplicates, 4103 records were screened in Rayyan, with 10% reviewed by two independent reviewers, achieving moderate inter-reviewer agreement (kappa = 0.44).^42^ Discrepancies were resolved through discussion with a second reviewer (LS). During title screening, 1517 irrelevant articles were excluded, and abstract screening eliminated 2133 additional records, leaving 453 for full-text review. Ultimately, five articles met the eligibility criteria and were included in this review [Table 1]. To ensure a comprehensive search, reference lists of included studies were reviewed, but no additional articles were identified. One study included university students and retired employees in the sample; however, it was retained due to the small size of the non-working group (*n* = 6) and the limited number of studies in this area. The study selection process is summarised in the PRISMA flowchart [Figure 1].

**Table 1.** Study and participant demographic characteristics.

Author, year	Participants	Female (%)	Employment status (%)	Sickness absence, Mean (SD)	Follow- up	Intervention	Control	Main outcome	Measuring instrument
Del Pozo-Cruz et al., 2012^43^	90 university office workers with subacute low back pain lasting 6–12 weeks; IG: *n* = 44 (mean age: 46.83 ± 9.13) CG: *n* = 46 (mean age: 45.50 ± 7.02)	IG = 84.8 CG = 88.6	Full-time employed (100%)	N/R	9 months	Daily email reminders during weekdays containing a link to the online sessions with pre-recorded exercise video demonstrations and postural education material	Standard preventive occupational care and medication	(1) Pain intensity (2) Risk of chronicity↓ (3) Functional disability (4) Quality of life (5) Back trunk muscle endurance	(1) Visual Analogue Scale (VAS) (2) Keele STarT Back Screening Tool (SBST) (3) Roland Morris Disability Questionnaire (RMDQ) (4) The European Quality of Life Questionnaire-5 dimensions-3 levels (EQ-5D-3L) (5) The ShiradoIto lumbar and abdominal tests
Almhdawi et al., 2020^44^	41 governmental office workers with chronic low back pain lasting over 3 months; IG: *n* = 21 (mean age: 40.48 ± 7.22) CG: n = 20 (mean age: 41.70 ± 6.35)	IG = 66.7 CG = 40.0	Full-time employed (100%)	IG = 4.14 (7.60) CG = 3.30 (6.47)	6 weeks	The mobile application ‘Relieve my back’ consisted of general advice and instruction, office-based stretching exercises for lower back and abdominal muscles, and four daily phone notifications promoting walking breaks, right posture, and exercise.	Placebo version mobile application with general nutrition advice not related to low back pain management	(1) Pain intensity (2) Functional disability (3) Quality of life (4) Sleep quality (5) Physical activity level (6) Mental health	(1) VAS, 11 point (2) Oswestry Disability Index (ODI) questionnaire (3) The 12-item Short-Form Health Survey (SF-12) (4) Pittsburgh Sleep Quality Index (PSQI) (5) the International Physical Activity Questionnaire (IPAQ) (6) Depression Anxiety Stress Scale (DASS)
Anan et al., 2021^45^	94 manufacture company engineers with remarkable musculoskeletal symptoms who reported to have frequent or almost always low back pain; IG: *n* = 48 (mean age: 41.8 ± 8.7) CG: n = 46 (mean age: 42.4 ± 8.0)	IG = 18.7 CG = 28.3	Full-time employed (100%)	N/R	12 weeks	A fully automated chatbot based on artificial intelligence (secaide, Version 0.9) programmed to send the users individualised messages with exercise instructions and tips on improving pain symptoms at a fixed time every day through the smartphone's chatting app (LINE).	Daily 3-minute exercises to prevent stiff shoulders and low back pain during break time.	(1) Pain intensity (2) Improvement of pain symptoms	(1) Subjective 5-point assessment scale (2) Battery of questionnaires designed for this study
Cimarras-Otal et al., 2020^46^	18 assembly line workers with chronic low back pain; IG = 10 (mean age: 42.25 ± 7.28) CG = 8 (mean age: 42.20 ± 5.59)	IG = 20.0 CG = 50.0	Full-time employed (100%)	IG = 3.8 (12.01) CG = 12.12 (34.29)	8 weeks	A mobile application with general exercise recommended by the American College of Sports Medicine (ACSM) and exercise adapted to the movement pattern of work activity	A mobile application only with general exercise recommended by the ACSM	(1) Pain intensity and interference (2) Functional disability (3) Spine function	(1) Brief Pain Inventory - Short Form (BPI-SF) (2) ODI questionnaire (3) The flexion relaxation (F/R) test measures kinematic parameters (angle and flexion or (4) bending speed)
Irvine et al., 2015^47^	597 employees from four companies (trucking, manufacturing, technology, and a corporate headquarters) with low back pain; IG = 199 (mean age: N/R) CG 1 = 199 (mean age: N/R) CG 2 = 199 (mean age: N/R)	IG = 58.3 CG 1 = 58.8 CG 2 = 62.8	Employed IG = 95.5% CG 1 = 96.0% CG 2 = 96.5%	N/R	4 months	A web-based online program ‘FitBack’ with a self-monitoring tool to track pain levels, a self-care activity picker containing text articles and videos on pain, pain management, ergonomics, and exercises based on the job type, and weekly emails with gain-framed pain self-care messages and prompts to return to the program.	CG 1 received weekly emails with links to 6 websites providing general educational resources for low back pain. CG 2 received emails only containing invitations to complete the self-assessment.	(1) Pain outcomes (frequency, intensity, and duration) (2) Pain interferences (3) Functionality and Quality of life (4) Productivity (5) Presenteeism (6) Planner behaviour (self-motivation, behaviour intentions, self-efficacy, attitudes toward pain and pain catastrophising)	(1) Battery of questionnaires designed for this study (2) Adapted scale from Multidimensional Pain Inventory Interference Scale (MPI) and the Interference Scale of the Brief Pain Inventory (3) The 9-item Dartmouth CO-OP scale (4) The 4-item Work Limitations Questionnaire (WLQ) (5) The 6-item Stanford Presenteeism scale Battery of questionnaires designed for this study (6) Battery of questionnaires designed for this study

N/R: not reported; IG: intervention group; CG: control group; Sickness absence: sick leave days due to low back pain from last year.

### Quality appraisal

**Figure 2.**
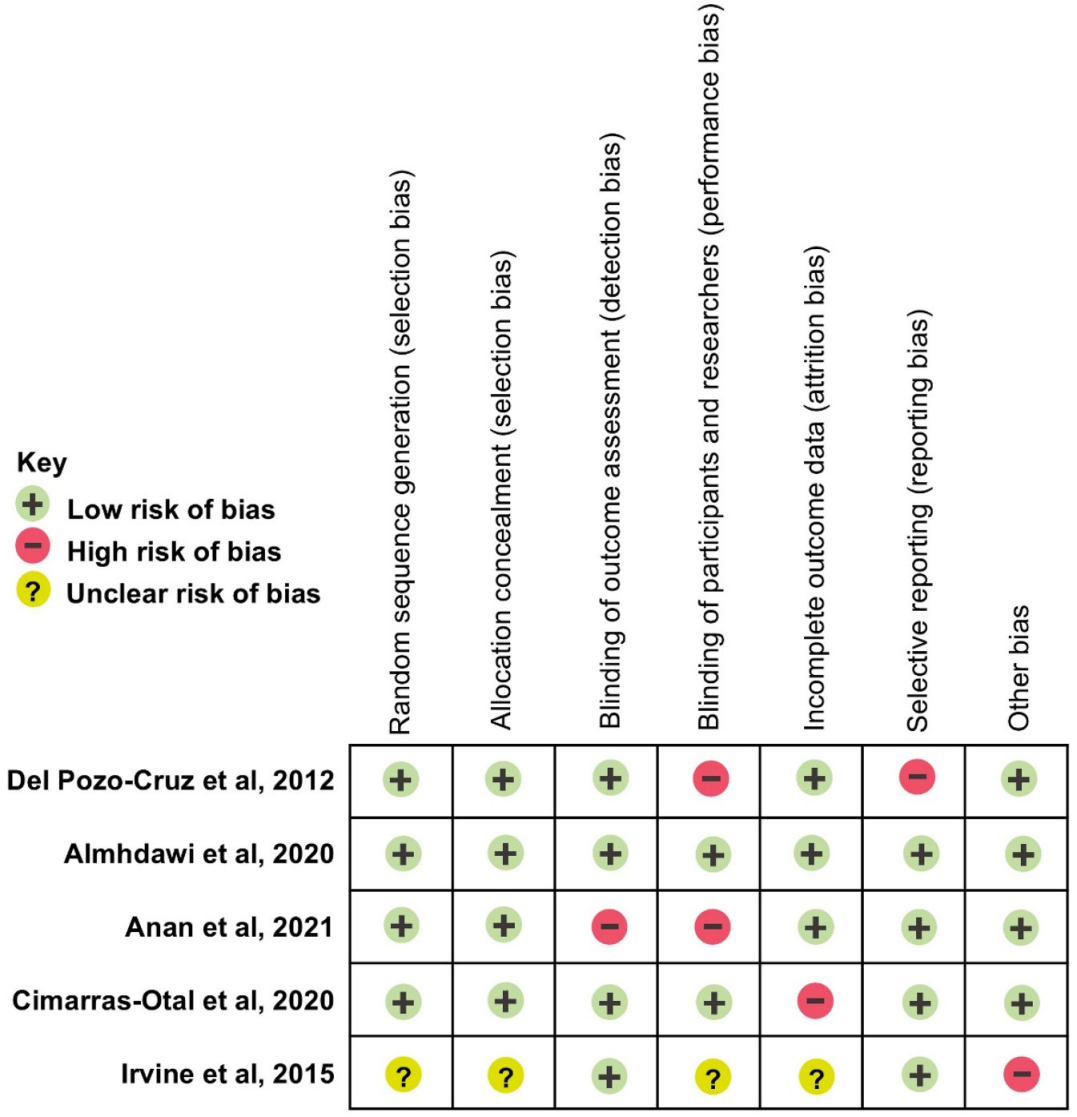
Risk assessment of bias in the included studies.

The quality of the five included trials was assessed using the Cochrane Collaboration's Risk of Bias tool.^41^ Overall, the studies demonstrated low to moderate quality, with notable variability in reporting practices [Figure 2].

Four trials used computer-generated random sequences for allocation,^43–46^ while one did not specify the method used.^47^ Allocation concealment was adequate in four trials but was not reported in one.^47^ Blinding practices varied. Two trials employed double-blind designs,^44,46^ one used single blinding,^45^ and two either lacked blinding or provided insufficient details.^45,47^ High attrition rates (55%) were observed in one trial,^46^ while another reported fraudulent recruitment practices and incomplete data reporting.^47^ Reporting bias was evident in one trial that inconsistently presented baseline and follow-up results.^43^ Four trials were funded by independent research grants, indicating a low risk of funding bias.^43–46^ One trial was funded by a healthcare technology company that developed the DHI under investigation, raising potential concerns about conflicts of interest.^47^ Additional risks included incomplete reporting of data security measures and unclear intervention details in some studies. Using the GRADE framework, primary outcomes such as pain intensity and disability were rated as moderate quality due to methodological limitations. Secondary outcomes, including psychosocial and work-related measures, were rated as low to very low quality because of variability in reporting. These findings underscore the need for more robust trials to evaluate DHIs for LBP management in workplace settings.

### Demographic data

The five RCTs included in this review were published between 2015 and 2021. Two were conducted in Spain,^43,46^ one in the USA,^47^ one in Japan,^45^ and one in Jordan.^44^ The trials lasted between 6 weeks and 6 months. A total of 840 participants were enrolled, with 322 allocated to intervention groups and 518 to control groups. Sample sizes ranged from 18 to 398 participants. Slightly more than half of the participants were female (56.3%), with individual studies reporting proportions ranging from 18.7% to 88.6%. Only one study^47^ reported demographic data on ethnicity, education, and income. Mean participant ages ranged from 40.48 to 46.83 years,^43–46^ except for one study that did not report age.^47^ All participants were employed, with employment rates ranging from 95.5% to 100%. Three trials took place in office settings,^43,44,47^ while two were conducted in manufacturing settings.^45,46^ Reported annual workdays lost to LBP ranged from 3.10 to 12.12 days in three studies.^44,46,47^

No studies reported the use of diagnostic imaging. One study included participants diagnosed with LBP by a physician within the past 3 months as an inclusion criterion.^46^ The remaining four studies included individuals with self-reported non-specific LBP, excluding those with a documented diagnosis of a specific pathology excluded.^43–45,47^ Eligibility criteria for LBP duration varied, with studies including participants with subacute LBP (4–12 weeks),^43^ chronic LBP (over 3 months),^44^ and those who had non-specific LBP within the past 3 months.^45–47^ Baseline levels of pain intensity, disability, quality of life, physical performance, and psychosocial outcomes across the five studies are presented in Table 2.

**Table 2.** Baseline pain-related participant characteristics.

Author, year	Pain intensity	Disability	Quality of life	Physical performance	Psychosocial outcomes
Del Pozo-Cruz et al., 2012^43^	100 mm Visual Analogue Scale, point IG: *n* = 44; Mean (SD) = 59.22 (11.96) CG: *n* = 46; Mean (SD) = 59.25 (11.38)	Roland Morris Questionnaire score, points IG: *n* = 44; Mean (SD) = 11.65 (2.14) CG: *n* = 46; Mean (SD) = 12.28 (2.63)	The European Quality of Life Questionnaire-5 dimensions-3 levels, point IG: *n* = 44; Mean (SD) = 0.78 (0.08) CG: *n* = 46; Mean (SD) = 0.75 (0.11)	The ShiradoIto Lumbar endurance test, s IG: *n* = 44; Mean (SD) = 77.52 (28.06) CG: *n* = 46; Mean (SD) = 77.17 (30.53) The ShiradoIto Abdominal endurance test, s IG: *n* = 44; Mean (SD) = 49.75 (31.11) CG: *n* = 46; Mean (SD) = 48.10 (32.16)	N/R
Almhdawi et al., 2020^44^	10 mm Visual Analogue Scale, point IG: n = 21; Mean (SD) = 5.62 (2.06) CG: *n* = 20; Mean (SD) = 5.10 (1.83)	Oswestry Disability Index questionnaire, point IG: n = 21; Mean (SD) = 30.95 (9.31) CG: *n* = 20; Mean (SD) = 31.05 (10.75)	The 12-item Short-Form Health Survey, point IG: *n* = 21; Mean (SD) = 61.05 (17.54) CG: *n* = 20; Mean (SD) = 64.21 (17.84)	The International Physical Activity Questionnaire, metabolic equivalent * minute/week IG: *n* = 21; Mean (SD) = 3438.5 (3464.8) CG: *n* = 20; Mean (SD) = 3706.5 (3161.3)	Depression Anxiety Stress Scale, point IG: *n* = 21; Mean (SD) = 22.95 (15.46) CG: *n* = 20; Mean (SD) = 17.21 (12.63)
Anan et al., 2021^45^	N/R	N/R	N/R	N/R	N/R
Cimarras-Otal et al., 2020^46^	Brief Pain Inventory - Short Form, point IG: *n* = 10; Mean (SD) = 4.2 (2.3) CG: *n* = 8; Mean (SD) = 5.0 (1.4)	Oswestry Disability Index questionnaire, point IG: *n* = 10; Mean (SD) = 17.0 (16.4) CG: *n* = 8; Mean (SD) = 16.7 (13.1)	N/R	The flexion relaxation test, angle IG: *n* = 10; Mean (SD) = 68.38 (9.47) CG: *n* = 8; Mean (SD) = 74.32 (13.89)	N/R
Irvine et al., 2015^47^	Questionnaires designed for this study, point IG: *n* = 199; Mean (SD) = 0.96 (1.26) CG1: *n* = 199; Mean (SD) = 1.22 (1.43) CG2: *n* = 199; Mean (SD) = 1.09 (1.34)	N/R	The 9-item Dartmouth CO-OP scale, point IG: *n* = 199; Mean (SD) = 20.41 (5.02) CG1: *n* = 199; Mean (SD) = 20.66 (4.74) CG2: n = 199; Mean (SD) = 21.01 (4.96)	N/R	The Patient Activation Measure-Short Form, point IG: *n* = 199; Mean (SD) = 3.14 (0.45) CG1: *n* = 199; Mean (SD) = 3.10 (0.47) CG2: *n* = 199; Mean (SD) = 3.09 (0.46) Tampa Scale of Kinesiophobia, point IG: *n* = 199; Mean (SD) = 2.25 (0.58) CG1: *n* = 199; Mean (SD) = 2.23 (0.61) CG2: *n* = 199; Mean (SD) = 2.22 (0.59)

N/R: not reported; IG: intervention group; CG: control group; SD: standard deviation.

### Intervention characteristics

The five included trials utilised diverse DHIs delivered via mobile applications (*n* = 3) or web-based platforms (*n* = 2). Trials conducted after 2020 predominantly employed mobile applications,^44–46^ while earlier studies used online websites.^43,47^ DHIs were used to deliver exercise programs, educational content, and in some cases, tailored interventions designed to enhance self-management of LBP in workplace settings. However, only two trials described tailoring processes, while the rest lacked detailed information on design and development [Table 3]. None of the interventions demonstrated any significant integration into existing occupational health pathways.

**Table 3.** Description of characteristics of digital health intervention in selected trials.

Author, year	Mode of delivery	Data collection	Theoretical framework	Classification model	Methods of tailoring	General intervention	Workplace-specific intervention
Del Pozo-Cruz et al., 2012^43^	Website	Paper-based self-assessment questionnaires collected by researcher	N/R	N/R	N/R	Type: Exercise Format: Video with audio instruction and written subtitles Duration: 7 minutes Frequency: 1 session daily over 9 months Target area: Main postural stability muscles (abdominal, lumbar, hip, and thigh muscles). Content: Exercise involving both extension and flexion movements Mobility exercises of large movements of the joints associated with postural stability muscles. Flexibility exercises using a static work methodology. Strength exercises of progressive shortening, stretching speed, and motion. Ratios (1:1, 1:2, 1:3, 2:1, 3:1), combined with slight isometric contractions of the muscles involved in the exercises. Moderate stretching of the muscles involved in the session.	Type: Education Format: Video with audio instruction and written subtitles Duration: 2 minutes Frequency: 2 sessions daily over 9 months Target: Sedentary behaviour Content: 1. Reminder of key postural issues 1. Advice on sitting posture, modifying the working environment, and choosing proper ergonomic equipment.
Almhdawi et al., 2020^44^	Mobile application	Paper-based self-assessment questionnaires collected by researcher	N/R	N/R	N/R	Type: Education and home-based exercise Format: Pictures and text instructions Duration: 20 minutes Frequency: Weekly 3–4 sessions over 6 weeks Target area: Back and abdominal muscles Content: Exercise: Strengthening exercises involving both extension and flexion movements across knees, deep abdominal, chest, arms, legs, and back. Education: Evidence-based information recommended by European clinical guidelines on: facts about your back; staying active/active rest; How to manage low back pain when having severe pain; managing pain medically; things you do that increase/decrease your low back pain; when to seek a physician (red flags); the importance of exercise.	Type: Office-based exercise and education Format: Pictures and text instructions Duration: 2 minutes Frequency: Weekly 3-4 sessions over 6 weeks Target: Lumbar spine Content: A reminder of key postural issues. Education: Improper body mechanics: wrong lifting; Proper body mechanics: correct lifting; Ergonomic workstation: correct body posture while working on a desk.
Anan et al., 2021^45^	Mobile application	Electronic self-assessment questionnaires were collected using an online automated system.	N/R	N/R	Users can choose their preferred exercise from a list of daily recommended exercises provided by the AI-assisted chatbot. Users can choose a time for receiving daily notifications by responding to the message sent by the AI-assisted chatbot.	Type: Education and exercise Format: Pictures and text instructions Duration: 3 min Frequency: At least 1 session daily over 12 weeks Target area: Shoulders and back Content: Detailed intervention content was not disclosed Exercise: Pre-designed stretching exercises. Education: Messages containing tips for daily activities to improve pain symptoms, and maintain good posture and mindfulness.	N/R
Cimarras-Otal et al., 2020 ^46^	Mobile application	N/R	N/R	N/R	N/R	N/R	Type: Exercise Format: Not disclosed Duration: Not disclosed Frequency: 3 times per week over 8 weeks Target area: Lumbar spine Content: Exercises designed based on 6 patterns of movement during work involving both extension and flexion movements, progressing with 3 levels: starting level (first 3 weeks), average level (fourth and fifth weeks), and advanced level (seventh and eighth weeks). Displacement in the workplace: Strengthening the muscle groups that were not active during the day. Cervical movement: cervical flexion or cervical rotation. Spinal movement: forward flexion or left and right rotation. Handle loads: cardiovascular exercise to improve motor control in case of performing excessive lumbar movement, such as raising, bringing near, or pushing loads. Range of shoulder movement: flexo-abduction improvement. Use of tools: grip strength training.
Irvine et al., 2015^47^	Website	Electronic self-assessment questionnaires were collected using an online automated system.	Social cognitive theory; theory of planned behaviour	Classify people with low back pain into 4 types based on primary job activity during the day: Sitters: sit most of the day. Standers: stand most of the day. Drivers: drive most of the day. •Lifters: do a substantial amount of lifting each day.	Activity picker: users can choose daily pain self-management activities from four modules: rest and relief, positive thinking, general fitness, stretching, and strength exercises for back pain. Self-monitoring: users can record daily pain intensity to produce a 7-day and 30-day graph of individual pain levels. Diary functions: Users can record their efforts and thoughts on pain management and self-management activities performed beyond the recommended activities on a daily basis. Progress report: Providing feedback to users based on their completion of recommended activities	Type: Education Format: Video with pictures and text instructions Duration: 1-4 minutes Frequency: Not disclosed Target: Pain prevention behaviour Content: Advice on: General aspects of pain and pain management. Cognitive and behavioural strategies to manage and prevent pain (e.g. controlling fear of pain, mindfulness, and relaxation, use of heat and ice, over-the-counter medications, benefits of staying active).	Type: Exercise Format: Video with pictures and text instructions Duration: 1-4 minutes Frequency: Not disclosed Target area: Pain prevention behaviour Content: Strength and stretching exercises Tailored by job type (sitter, stander, driver, and lifter). Detailed intervention content was not disclosed.

N/R: not reported; AI: artificial intelligence.

### Exercise

All five trials included exercise as a key intervention component, though the presentation and specificity varied. Two studies delivered exercises via pre-recorded videos,^43,47^ and two used text and images.^44,45^ One study did not specify the method used to demonstrate exercises.^46^ Three studies incorporated exercise management with intervention durations ranging from 6 weeks to 9 months.^43–45^ Three DHIs included exercises targeting key muscle groups, such as the abdominal, lumbar, hip, and thigh muscles^43,44^ as well as the lumbar spine^46^ utilising both flexion and extension movements. The remaining two studies referenced evidence-based exercise guidelines, including recommendations from the American College of Sports Medicine (ACSM), providing a set of stretching exercises with illustrated instructions and advice against maintaining a single position for over 30 minutes.^44,46^

### Education

Educational content was another core component of the DHIs in four trials, covering various topics such as ergonomics, sitting posture, pain anatomy and physiology, work-related mechanics, cognitive-behavioural strategies, and lifestyle changes.^43–45,47^ Education was delivered through videos (*n* = 2)^43,47^ or text-based content (*n* = 2).^44,45^ Only one trial described the development of its educational content as evidence-based, referencing European clinical guidelines on LBP.^44^

### Tailoring to individual needs

Two studies explicitly tailored interventions.^45,47^ Irvine et al. developed ‘FitBack’, a workplace DHI for LBP self-management categorising participants into job-based categories (sitters, drivers, lifters, and standers) and tailoring exercises and education accordingly. The framework, based on Social Cognitive Theory and the Theory of Planned Behaviour, allowed self-monitoring and module selection (Rest, Positive Thinking, Health Literacy, and Exercise), through classification algorithms that were undisclosed and unvalidated. Anan et al. used an AI-driven chatbot delivering tailored exercise interventions, allowing participants to select exercises and schedule messages, but did not clarify whether tailoring considered LBP characteristics or occupational demands.

While tailoring is suggested to enhance engagement and intervention relevance, its impact on outcomes remains unclear due to limited and inconsistent reporting. The remaining three studies provided generic interventions without tailoring them to individual needs. However, four studies incorporated work-specific intervention content, including ergonomic education addressing sedentary behaviour,^43^ spine resilience in the workplace,^44^ and exercises tailored to work activities^46^ and occupation type.^47^

### Clinical outcome data

Clinical outcomes in the included studies were diverse, reflecting the multifaceted nature of LBP. Pain intensity was assessed in all five trials, with most using the Visual Analogue Scale (VAS),^43–45^ though one study did not specify the measurement tool.^47^ Disability was evaluated in three trials using the Roland-Morris Disability Questionnaire (RMDQ)^43^ and the Oswestry Disability Index (ODI).^44,46^ Risk of chronicity was reported in one study using the STarT Back Screening Tool (SBST).^43^ Quality of life was measured in three studies using validated instruments such as the European Quality of Life 5 Dimensions 3L (EQ-5D-3L),^43^ the Dartmouth Collaborative Primary Care Information Project (CO-OP),^47^ and the Short-Form 12 (SF-12) Health Survey.^44^ Physical performance was assessed in three trials, with objective laboratory-based measures evaluating lumbar endurance^43^ and spinal mechanical function,^46^ while one trial used the International Physical Activity Questionnaire (IPAQ).^44^ Psychosocial outcomes were reported in two studies, measuring factors such as depression, anxiety, stress, and pain catastrophising using tools like the Tampa Scale for Kinesiophobia (TSK),^47^ the Patient Activation Measure (PAM), and custom-designed questionnaires for self-efficacy and behavioural intentions.^47^ Work performance was assessed in a single trial, utilising the Work Limitations Questionnaire (WLQ) and the Stanford Presenteeism Scale (SPS-6).^47^ These outcomes highlight the broad range of measures used to capture the impact of DHIs on both clinical and workplace-specific aspects of LBP.

### Intervention effects

Of the five trials, one study reported no adverse health effects of the intervention.^43^ The remaining four studies did not disclose information on adverse events.^44–47^ Three studies reported participant adherence, with dropout rates in the intervention group ranging from 4.5% to 8%.^43,44,47^ Two studies reported high dropout in both the intervention (21.3%–50%) and the control groups (23.3%–60%).^45,46^ Only one study reported data on DHI usage, with an average of 6 minutes and 40 seconds per day.^44^

### Pain intensity

All five included trials assessed pain intensity as a primary outcome, with three using VAS of varying lengths.^43–45^ All trials reported a reduction in pain intensity, with three demonstrating statistically significant improvements.^44,45,47^ Irvine et al. reported a small and non-significant intervention effect for the tailored DHI compared to a non-tailored DHI (*η*² = 0.008, *p* = 0.266). The specific measurement tool for pain intensity was not disclosed in this trial.

### Disability

Three trials identified LBP-related disability as a primary outcome. Two studies used the ODI,^44,46^ and one employed the RMDQ.^43^ Del Pozo-Cruz et al. reported significant reductions in disability scores for participants using a DHI compared to standard occupational care (MD = –8.42, 95% CI: −9.76 to −7.07, *p* < 0.001). However, Cimarras-Otal et al. found no significant differences between groups using a DHI and a placebo DHI with ACSM-guided exercises (MD = –0.26, 95% CI: −1.81 to 1.29).

### Quality of life

Three studies assessed the quality of life using validated tools: the EQ-5D-3L,^43^ the SF-12,^44^ and the CO-OP.^47^ Almhdawi et al. reported significant improvements in physical health-related quality of life. Irvine et al. demonstrated enhanced quality of life improvements with a tailored DHI compared to a non-tailored DHI (*η*²= 0.029, *p* =0.003).

### Physical performance

Three trials evaluated physical performance. Laboratory-based assessments in two trials found significant improvements in lumbar muscle endurance^43^ and spinal mechanical function.^46^ However, Almhdawi et al. reported no significant differences in physical activity levels, measured using the IPAQ. The authors did not specify whether they used the IPAQ short or long form and presented their findings as metabolic equivalents rather than categorical activity levels.

### Psychosocial outcomes

Two trials investigated psychosocial factors such as stress, anxiety, depression, and pain catastrophizing.^44,47^ Irvine et al. observed significant benefits from the tailored DHI in self-efficacy, behavioural intentions, and a sense of responsibility for self-management. However, no significant differences were reported in pain catastrophizing (measured using the TSK). Almhdawi et al. found no group differences in sleep quality or mental health outcomes between a work-based DHI and a placebo DHI.

### Risk of chronicity

Only one trial measured the risk of chronic LBP using the SBST.^43^ This study found a significant increase in the proportion of participants classified as low risk of chronicity (37%, *p* = 0.005) after 9 months of DHI use compared to standard occupational care.

### Work performance

Only one trial evaluated work-related outcomes, using the 4-item WLQ and the SPS-6.^47^ This study found no significant differences in work productivity or presenteeism between participants using tailored and non-tailored DHIs.

## Discussion

This SR evaluated the effectiveness of workplace-based DHIs that support self-management of LBP on health and work outcomes, examined how these interventions are tailored to individual and occupational needs, and assessed their integration with workplace health pathways. Five randomised control studies met the inclusion criteria, evaluating pain intensity, disability, and physical performance as primary outcomes, and psychosocial factors, quality of life, and work-related measures such as sickness absence and presenteeism as secondary outcomes. Follow-ups ranged from 6 to 9 months, with three studies providing short-term follow-ups (6–12 weeks)^44–46^ and two offering mid-term (4–9 months).^43,47^ DHIs were delivered via web-based platforms^43,47^ or mobile apps,^44–46^ primarily through educational materials and exercise instructions in text, images, or videos. Four trials incorporated workplace-specific content, including exercises tailored to work activities^46,47^ or environments,^44^ and ergonomic workplace education.^43^

Evidence from the included trials suggests DHIs that support self-management of LBP provide low to moderate benefits for pain intensity, disability, and physical performance. This is consistent with previous reviews of DHIs for musculoskeletal conditions in clinical settings highlighting the effectiveness of combining education and exercise.^29,30^ Rathnayake et al. similarly reported moderate improvements in pain and disability in chronic LBP. However, the impact on quality of life, psychosocial factors, and work performance was mixed. One trial found improvements in self-management motivation and pain attitudes,^47^ while another reported no significant effects on depression, anxiety, or stress.^44^ These inconsistencies reflect broader literature that DHIs often fail to adequately address the complex psychosocial aspects of LBP.^48,49^

There was no consistent evidence of direct integration of DHIs into workplace health pathways, as interventions primarily focused on individual-level self-management rather than alignment with occupational health frameworks. None of the studies incorporated employer-driven occupational health services or policies, despite their recognised importance in the implementation and effectiveness of such interventions.^50^

A recent SR by Blake et al. highlighted the benefits of workplace-based DHIs that support self-management of LBP on chronic pain management and access to work-related advice.^28^ Integrating DHIs into occupational health systems could help organisations overcome barriers to early LBP self-management and provide a proactive approach to reducing absenteeism, ultimately easing the burden on healthcare services.^51,52^ Additionally, broader stakeholder engagement in the integration process is crucial to ensuring that DHIs meet the needs of working populations, enhancing their real-world impact. Underpinning designed interventions with appropriate theory and a logic model to guide the evaluation of health and work-related outcomes, including absenteeism and presenteeism, will be essential for advancing this field.^53^

Despite some promising outcomes, the included studies presented considerable variability in participants’ job roles, demographics, and baseline LBP characteristics. Definitions of LBP vary considerably across studies, with pain durations ranging from 6 weeks to 9 months. Such inconsistencies complicate direct comparisons and generalisations, as individuals of different stages of pain may respond differently to DHIs. Heterogeneity also extended to methodological quality, with substantial variation in intervention content, outcome measures, and control group designs. Only two studies explicitly reported evidence-based DHI development,^44,46^ and few provided comprehensive details on intervention components. These reporting gaps, previously noted in systematic reviews, limiting the identification of effective workplace DHIs for LBP management.^27,30^ This aligns with broader critiques of DHI research, particularly in the lack of transparency, standardisation, and validation of tailoring models.^54^

Tailored interventions were underrepresented, with only one trial^47^ meeting the criteria by classifying participants based on job types and preferences. However, methodological weaknesses, including unvalidated outcome measures and potential funding bias, reduced its reliability. Validating classification models and tailoring algorithms is particularly crucial for improving the effectiveness of tailored interventions. The promising clinical effectiveness and adherence seen in recent AI-based DHIs that support self-management of LBP suggest that AI may enhance classification accuracy for better-tailored interventions.^55–57^ Workplace LBP data could play a key role in training AI models, while AI-based DHIs also offer the potential for enhancing organisational-level LBP management. The high accuracy of a recent AI-driven LBP classification model by Liu et al. highlights its potential advancing tailored workplace interventions by personalising ergonomics and exercise programmers based on an individual's spine function signature.^58^

Workplace-specific interventions often perform differently across occupations due to variations in physical demands, ergonomic risks, and resource access,^16,59^ highlighting the need for context-sensitive approaches. The inconsistent tailoring methods across studies further reflect broader gaps in standardised intervention design and evaluation.^29,60^ Future research should adopt standardised frameworks, such as the Medical Research Council (MRC) framework for complex health interventions,^53^ to ensure rigor in DHI development and evaluation.

Certain limitations hinder interpretation, including short follow-up durations, limiting understanding of long-term effects, and insufficient stakeholder involvement, which could enhance intervention relevance and feasibility. While imaging is not routinely recommended for uncomplicated LBP diagnosis,^17^ its absence may obscure the identification of specific underlying mechanisms. These limitations underscore the need for more uniform inclusion criteria and the potential incorporation of objective classification tools in future research, to enhance comparability and better delineate the effectiveness of DHIs supporting self-management in LBP populations. Additionally, the lack of detailed intervention descriptions and development processes highlights the need for comprehensive reporting, including Supplemental Material. Future research should therefore prioritise standardised frameworks, occupational health collaboration, and longer follow-ups to assess levels of integration and sustained impacts. Validating tailoring methods and aligning DHIs with workplace policies are essential for maximising their effectiveness in workplace LBP management.

## Conclusion

Despite the limited research on DHIs that support self-management for workplace LBP, this systematic review suggests their potential to improve pain intensity, disability, and physical performance. However, their effectiveness on psychosocial factors, quality of life, and work-related outcomes (e.g., sickness absence and presenteeism) remains inconsistent, likely due to a greater focus on individual rather than organisational-level factors, limited tailoring to individual and occupational needs, and minimal integration into workplace health systems. Addressing these gaps through validated tailoring models, standardised intervention frameworks, and stronger alignment with occupational health policies is essential to enhance scalability, engagement, and long-term impact in workplace LBP management.

## Supplemental Material

sj-docx-1-dhj-10.1177_20552076251336281 - Supplemental material for Systematic review of digital health interventions to support self-management of low back pain in the workplace Supplemental material, sj-docx-1-dhj-10.1177_20552076251336281 for Systematic review of digital health interventions to support self-management of low back pain in the workplace by Minghao Chen, Valerie Sparkes and Liba Sheeran in DIGITAL HEALTH

sj-docx-2-dhj-10.1177_20552076251336281 - Supplemental material for Systematic review of digital health interventions to support self-management of low back pain in the workplace Supplemental material, sj-docx-2-dhj-10.1177_20552076251336281 for Systematic review of digital health interventions to support self-management of low back pain in the workplace by Minghao Chen, Valerie Sparkes and Liba Sheeran in DIGITAL HEALTH
