# Supplementary material for: Systematic review of digital health interventions to support self-management of low back pain in the workplace
Source: Digit Health. 2025 May 26;11:20552076251336281. doi: 10.1177/20552076251336281 (PMC12117239; doi:10.1177/20552076251336281)
Supplement: sj-docx-2-dhj-10.1177_20552076251336281 - Supplemental material for Systematic review of digital health interventions to support self-management of low back pain in the workplace [file sj-docx-2-dhj-10.1177_20552076251336281.docx]

**Appendix 2.** The 3-arms search strategy for database searching

**1.Low back pain**

exp Back pain/

(back pain* OR backache*).tw.

((spin* adj2 pain*) OR (lumbar adj2 pain*) OR (pelvi* adj2 pain*) OR (thoracic adj2 pain*)).tw.

**2. Digital Health Intervention**

(Web-based intervention* OR Internet-based intervention* OR Online intervention*).tw. (Digital adj2 intervention*).tw.

(Digital health* OR mHealth* OR eHealth* OR e-health* OR Mobile health*).tw.

Exp Internet/

(Web* OR Website* OR Web site* OR Webpage* OR Web page* OR Online*).tw.

Exp Computers/

Exp Software/

Exp Mobile applications/

(App* OR Online application* OR App* OR Internet-based application* OR Computer-based application*).tw.

Exp smartphone/

(Smart phone* OR iPhone* OR android* OR Mobile device*).tw.

Exp Wearable Electronic Devices/

**3. Workplace**

Exp Occupational Health/

(Occupational Wellness* OR Occupational Wellbeing* OR Occupational well-being* OR Occupational Safety* OR Occupational setting* OR Working Conditions* OR Work Environment*).tw.

Exp Workplace/

(Factory* OR Company* OR Office* OR Warehouse* OR Industr* OR Worksite* OR Organisation* OR Organization* OR business* OR workspace* OR workstation* OR Work Activities*).tw.

Exp Work/

Exp Occupations/

(Employe* OR employment* OR Worker* OR Staff* OR career* OR job* OR labor* OR labour*).tw.
